# From school strikes to webinars: Mapping the forced digitalization of Fridays for Future’s activism during the COVID-19 pandemic

**DOI:** 10.1177/13548565221148112

**Published:** 2022-12-24

**Authors:** Giuliana Sorce, Delia Dumitrica

**Affiliations:** Eberhard Karls University Tübingen, Germany; 6984 Erasmus University Rotterdam, Netherlands

**Keywords:** Digitalization of activism, repertoires of action, social movements, tactical transformation, Fridays for future, COVID-19, qualitative research

## Abstract

This paper discusses the forced digitalization of activism brought about by the COVID-19 pandemic in the case of the transnational environmental youth movement Fridays for Future (FFF). Theoretically, we engage with social movement action repertoires to study the shifts in protest tactics associated with the social restrictions during the early stages of the pandemic. A qualitative content analysis of 781 posts across all 27 national FFF Facebook pages in the European Union reveals four clusters of digital action types: digital contentious actions; online information and education; digital community engagement and online partnership development. While digital media were part of FFF’s action repertoire in pre-pandemic times, our findings yield that the shift from the movement’s iconic street protests to exclusively digital tactics privileges community-building and education over contentious actions, potentially softening the political impact of the movement’s landmark ‘school strike’. Furthermore, although timely tactical flexibility kept the movement going during country lockdowns, the forced digitalization in the early stages of the pandemic primarily recombined existing action tactics rather than innovating them.

On the day COVID-19 was declared a pandemic, Greta Thunberg, Swedish activist and central figure of the transnational youth climate movement Fridays for Future (FFF) communicates to her social media followers that “experts urge us to avoid public gatherings for a better chance to #flattenthecurve and slow the spreading of the Coronavirus” (11 March 2020). Thunberg later updates her pinned Tweet to encourage protesters to join digital strikes instead. In response, national FFF collectives swiftly announced the move of all of their face-to-face collective actions to the online space, marking the beginning of the forced digitalization of the movement’s flagship tactic, the ‘school strike’, in response to the COVID-19 pandemic.

FFF has emerged as an important factor in the contemporary transnational environmental movement scene with a particularly large following in the European Union (EU). With its ‘future’ narrative, FFF has been successful in mobilizing youth, a social layer traditionally seen as politically passive. To flag the urgency of the climate crisis, FFF followers skip school to protest. School strikes have historically been ambivalent forms of collective action that seek attention through rule-breaking; to cease this withdrawal, strikers demand political action. In pre-pandemic times, FFF collectives used a digitally-supported action repertoire that relied mainly on advertising for participation in their analogue events. Digital media, such as websites, social media platforms or mobile apps were used to communicate with adherents and mobilize for participation but were not used as a main site for activism. In 2019 alone, over 17,000 coordinated weekly protests have drawn 7.3 million strikers to the streets worldwide (Teune, 2020: 134), making FFF a movement that neither politics nor media can ignore.

Recently, FFF has begun to attract scholarly attention. Studies have examined the mobilization patterns of strikers (Wahlström et al., 2019), media coverage of protests (Ryalls and Mazzarella, 2021; von Zabern and Tulloch, 2021), or Greta Thunberg as a leadership figure (Olesen, 2020; Sorce, 2022). In relation to the pandemic, Haßler et al. (2021) found that the German FFF Twitter account saw a decline in activity during the first wave of the pandemic; yet, they argue, FFF managed to generate attention through online protests. Similarly, Sorce and Dumitrica (2021) have studied how FFF has rhetorically incorporated the COVID-19 pandemic into their digital protest communication, noting the importance of social media to keep followers invested in the cause. Indeed, de Moor et al. (2021: 1) argue that during the pandemic, ‘FFF climate protests have continued online’, which ‘arguably marks the end of the first chapter of the recent climate protest cycle’. This paper picks up here to ask how FFF has engaged in tactical adaptation during the pandemic, paying particular attention to the role of social media as the only permitted site for activism during the first Europe-wide lockdown period of 2020. As with other contemporary movements, FFF had previously integrated social media in their information, communication and mobilization practices (Boulianne et al., 2020; Haßler et al., 2021). However, this integration primarily supported and enhanced analogue protesting during the movement’s flagship action – the school strikes.

Empirically, the multi-sited case study employs a qualitative content analysis of *N = 781* social media posts gathered from the 27 official FFF country pages in the EU on Facebook – the movement’s most commonly used social media platform. Via two rounds of coding, this paper identifies four clusters of digital actions present during the intensified digitalization of FFF’s activism in the early stage of the pandemic: digital contentious actions; online information and education; digital community engagement; and online partnership development. Categorizing these action types renders the effects of FFF’s forced digitalization of activism visible: While digital versions of the iconic Friday school strike in the streets across Europe provided adherents with opportunities to perform as digital strikers, this online action also shifted the activist focus from contention to collaboration. We find that the early stages of the pandemic, the movement’s use of social media remained focused on informational and educational resources, community-building, and, to a lesser extent, on partnership development. While our case cannot capture the overall impact of the COVID-19 pandemic on social movements, it responds to recent calls to map the impact of forced digitalization on the contentious work of social movements during crises (Della Porta, 2022). Further questions remain on how the new digital protest cycle might permanently transform FFF’s activist repertoire and whether other movements have undergone similar digital transformations.

## Repertoires of action and digitalization

We approach FFF as one of the newly established and highly mediatized collectives in contemporary environmental movements. FFF seeks to effect environmental policy changes by raising awareness against climate change. Since its origination in 2018, FFF represents a decentralized network of locally formed youth collectives inspired by Greta Thunberg’s charismatic figure (Boulianne et al., 2020). While connected via the FFF International hub (featuring a Web site and several social media channels), FFF grassroots collectives self-organize school strikes and act within their respective local communities (see also Pickard, 2019).

When the COVID-19 pandemic struck in early 2020, FFF quickly adapted its repertoire of action by going completely digital. While the movement had already relied on digital media prior to the pandemic, the abrupt introduction of social restrictions across Europe meant that the street strike and all other face-to-face activities could no longer take place. We refer to this protest cycle as “forced digitalization” to examine how the different FFF national collectives dealt with it pandemic lockdown regulations.

Social movements’ repertoires of action refer to collectively shared understandings of what constitutes legitimate types of actions against a political opponent. Such actions may include protests, boycotts, sit-ins, flash-mobs but also digital action in the forms of petitions or email bombing. As ‘culturally resonant forms of action’ (Tarrow, 2011: 16), repertoires matter to stakeholders and adherents. Specific contentious action tactics, such as the school strike, ‘improvise on shared scrips’ (Tilly, 2006: 35) that become recognizable, even expected by followers.

In practice, repertoires remain fluid and can evolve, though usually, ‘innovation occurs incessantly on the small scale’ (Tilly, 2006: 35) Changes in the political opportunity structures, for instance, can spur transformations in movement’s repertoires of action. Movements also learn from one another, adopting and transforming existing (successful) forms of action (Tarrow, 2011). Finally, changes in the media and communication opportunity structure (Cammaerts, 2012) can also create openings for tactical transformation; however, tactical transformation and innovation is inherently dialectical, simultaneously advancing and constraining movements.

Over the last decade, digital media have become seamlessly integrated into movement action repertoires, affording organizers more control of their own messages, opportunities to amplify their cause to wider audiences and diversify participation. For civil society organizations, Lovejoy and Saxton (2012) note that social media hold communicative functions for the dissemination of information, creation of an online community and a space to mobilize for action. The internet also provides new avenues for contentious action (Van Laer and Van Aelst, 2010), potentially lowering the cost of physical activism. In creating a space for digital political participation, ‘slacktivist’ involvement (such as posting on Facebook or signing an online petition) can become politically significant. Thus, online tactics can become direct social actions that ‘circumvent the traditional state-addressing repertoires of action and focus instead on a ‘self-changing’ society as part of everyday politics’ (Bosi and Zamponi, 2015: 368–369).

Furthermore, social media can also bring visibility to a ‘new model of flash mobilization in which collective action is so inexpensive that small time and content investment of participants allow many individuals to participate quickly’ (Theocharis et al., 2015: 204). In a related line of argumentation, the affordances of digital networks have been credited with the ease of movement formation and action (Bennett and Segerberg, 2012). The connective action approach sees social media platforms as enabling individuals to come together and perform a networked movement without necessarily sharing a collective identity.

Such arguments, however, are not without limitations. Social movements require sustained, collective and contentious actions that bring together various types of social actors (Rucht, 2017). This type of work entails complex communication processes and digital media become interwoven into movements’ hybrid communication ecosystems (Chadwick, 2013). Such ecosystems enable movement actors to engage in the different types of action (collective identity formation, mobilization, organization, amplification, contention, etc.) necessary for achieving political impact (Treré, 2019). Social media may offer opportunities for ‘creative participation’ in protest action (McFarland, 2010); however, their resonance as ‘culturally resonant forms of action’ (Tarrow, 2011: 16) and specifically, their political impact, remain widely debated (see also Khazraee and Losey, 2016) Furthermore, digital activism is intrinsically embedded in corporate capitalism, algorithmic control and surveillance (Dencik and Leistert, 2015). These structures limit digital activism in terms of practices, visibility and contentious effects.

While digitalization may offer new avenues for tactical transformation, it does not, in and of itself, ‘replace traditional forms of activism and face-to-face communication’ (Van Laer and Van Aelst, 2010: 1164). Indeed, tactical transformation often involves adaptation and recombination of existing actions rather than innovation (McCammon, 2008). Tactical innovation seems to hinge on tactical flexibility (McCammon, 2003) as to produce new tactics (McAdam, 1983) or recombinations (Wang and Soule, 2016) relevant to a specific context, such as a crisis. Yet, resulting tactics can also ‘shift the internal organization of a movement, as well as its external interactions in public and political arenas’ (Galli, 2016: 260). A movement’s success with transformed tactics hinges on how well organizers manage adaptation and how well stakeholders and adherents respond (see also McCammon, 2003). Thus, a ‘new, “digitalized” social movement repertoire of collective action’ (Van Laer and Van Aelst, 2010: 1148) emerges as part of a movement’s overall media ecosystem and often, within the scope of existing tactics.

For the case of the COVID-19 pandemic, della Porta (2022) notes that activists employ a plethora of new technologies, actively harnessing the power of online interaction to build contention for their cause. In offering the digital strike as a hallmark action by social movements in pandemic times, she calls FFF’s global digital strike the most notable one. In this paper, we approach the forced digitalization of FFF’s action repertoire as a contingent and contextual transformation brought on by the COVID-19 pandemic. Theoretically then, this study affords an opportunity to understand how movements digitalize their actions and how tactical transformation occurs. In turn, mapping the digital action repertoire allows insights into how new media practices during crises change a movement’s protest dynamics, including potential new areas of emphasis beyond physical flagship actions.

## Method

To capture the forced digitalization of FFF’s action repertoire across its decentralized network on social media, this case study analyzes the 27 public Facebook pages from EU collectives. National collectives serve as hubs for the local chapters in their respective countries and as country nodes for the transnational movement. We analyze the early stages of FFF’s going digital with a focus on their Facebook communication. National FFF collectives usually have their own websites and use several social media platforms, with Facebook, Twitter and Instagram being the most common ones across the EU. The majority of national groups also offer instant messenger communication via WhatsApp or Threema as a channel to receive updates on group activities. While FFF’s communication ecosystem extends beyond Facebook and commonly includes Instagram and Twitter, Facebook is the most commonly used social medium of FFF collectives in Europe. Indeed, we noted that content posted on Facebook was available publicly, richer in text, and commonly shared to Twitter and Instagram in abbreviated versions. Furthermore, posts from Facebook pages provided a richer corpus than websites, as many were not updated regularly and thus did not capture daily updates on movement activities.

Our sample thus includes all Facebook posts by all European national collectives beginning with the first update about the Corona crisis (12 March 2020) up to the first Global Digital Strike event (24 April 2020). In this study, a national Facebook page (*N = 27*) forms the unit of observation is a while a post (*N = 781*) constitutes the unit of analysis. Data gathering and analysis was enabled by our multinational research team’s diverse foreign language proficiency.^1^ During this six-week sampling period, all national collectives (except Ireland, whose page seems abandoned), moved their activism to social media and tactics of actions began to stabilize. Table 1 maps the national collectives under study along with relevant metrics.

**Table 1.** Country overview and sampling metrics.

Country	Followers	Posts	Maximum engagement (Singular post)
Austria	1599	33	357
Belgium	36327	99	325
Bulgaria	4233	30	67
Croatia	2936	11	34
Cyprus	2568	13	677
Czechia	11367	28	951
Denmark	7605	25	305
Estonia	2317	28	175
Finland	1984	10	135
France	19811	11	80
Germany	101912	45	2924
Greece	3366	7	516
Hungary	13558	41	916
Ireland	1279	0	0
Italy	41439	57	1327
Latvia	1570	26	41
Lithuania	3037	34	96
Luxembourg	2986	5	47
Malta	249	4	1
Netherlands	2555	2	56
Poland	36718	35	1598
Portugal	8343	32	95
Romania	5670	19	136
Slovakia	3562	30	249
Slovenia	6793	24	847
Spain	5253	15	123
Sweden	9566	28	579
	338603	781	12656

A qualitative content analysis helped us gather and systematize the specific aspects of FFF’s forced digitalization. An important distinction to its quantitative counterpart, this qualitative approach allows structuring and systematizing information rather than transforming it numerically (Kuckartz, 2014). With the Facebook posts as our unit of analysis, we analyze the meaning-making practices of digital communication practices in the context of a social movement. Hence, we payed attention to the multimedia forms of Facebook posts, including text, images, memes and (external) links. In particular, we focused on the communicative function of the posts, that is, the type of action that a post solicits of its readers.

To identify these communicative functions, we employed an analytical procedure using a mix of deductive and inductive coding. We began by going through each country’s FFF Facebook page, recording the different functions of the posts in an Excel spread sheet (e.g. mobilize for action, provide information, etc.). This first reading allowed us to identify several themes: announcements for digital events and online campaigns along with asynchronous and synchronous opportunities to engage with organizers and read media content (in-house and external).

In the second stage, we reviewed our data systematically by developing a coding frame. Theoretically informed by previous analyses of social media in organizing (Lovejoy and Saxton, 2012) and internet-supported action repertoires (Van Laer and Van Aelst, 2010), we found 33 tactics of action (e.g., boost morale, sign an online petition, partake in a webinar) that formed our codes. We subsequently abstracted these codes into four higher-order categories: digital contentious action, online community engagement, digital information and education provision, and online partnership development. To deal with ambiguity, we focused on the contextual purpose of the post – for instance, a hashtag could be intended as group signaling, event branding or a call for action. Here we used the contextual use of the element to assign this code to a single category. In this example, the #stayathome hashtag was used in posts that announced the cancellation of physical school strikes to ask for participation in digital alternatives. Thus, this tactic (hashtag use) fit best into the ‘digital contentious actions’ category. A final reading of all Facebook posts ensured that the codes fit into only one category.

## FFF’s digitalized action repertoire

As a youth movement, FFF has been employing digital communication to engage with followers on social media prior to the pandemic. Indeed, Haßler et al. (2021) found that before the country lockdown, ‘the volume of tweets was substantially larger during big protest events as compared to non-event days’ (18). A cursory look at the public pages under study suggests pre-COVID-19 Facebook posts mainly announce strike logistics or promote other physical events. In that sense, our findings here echo the claim that the pandemic has fundamentally affected the movement’s action repertoire and inaugurated a new cycle of protest.

Beginning in mid-March 2020, the majority of national FFF media channels featured a formal announcement that the Corona crisis brings the physical school strikes to a halt. In compliance with social distancing and public assembly restriction measures, FFF Facebook pages around Europe featured posts such as

On Friday, 13.03., the big demonstration… in Helsinki with its parade will be cancelled due to the threat of the spreading Coronavirus (FFF Suomi, 12 March 2020)

Join our #climatestrikeonline. We fully understand the current context and do not want to contribute to the spread of COVID-19… Even if we won’t be gathering on the street for a while, we move the protests online (FFF Romania, 13 March 2020)

**Figure 1.**
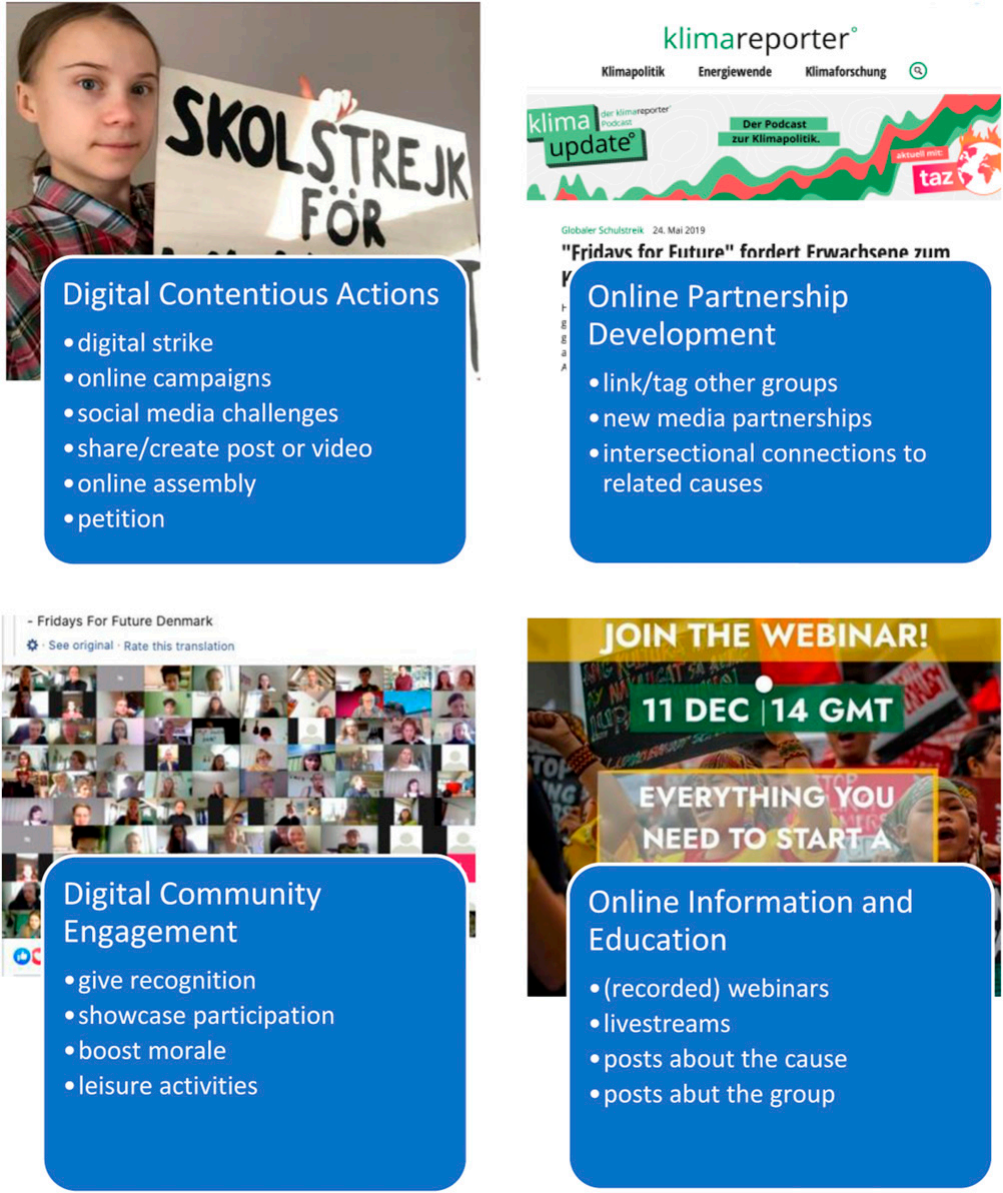
FFF’s digitalized activist repertoire in Europe during the COVID-19 pandemic.

In the weeks following the online shift, we see FFF’s newly digitalized action repertoire unfold in four clusters of action types. Figure 1 visualizes our findings and offers an overview of FFF’s repertoire of action under the COVID-19 restrictions. In what follows, we describe the four main movement actions on Facebook and illustrate them with examples from the data corpus.

### Contentious action

#### Digital strikes

With the exception of Malta, Ireland, Netherlands and Luxembourg, whose Facebook pages generally were not very active, all FFF national collectives advertised ‘digital strikes’ as *the* replacement for the iconic school strikes in the streets. Such ‘digital strikes’ were, however, of a slightly different nature than the pre-pandemic school strikes: they did not entail any withholding of time from school or work, as the word ‘strike’ may indicate. Instead, organizers instructed followers to take a picture with a protest sign, set it as their profile picture, and use hashtags such as #climatestrikeonline or #digitalstrike. Some national collectives sought to build up the momentum for these events by first announcing the digital strike events, then reminding followers throughout the week and finally thanking those who participated.

Four FFF collectives tapped into the synchronous possibilities of online activism to get groups of followers together at the same time, turning their digital strike events into livestreams (Slovenia, Poland, Lithuania and Hungary). This allowed followers to experience the digital replacement as a collective action event. Livestreams were also used as an opportunity to inform followers about digital strike logistics (Austria, Finland, France, Latvia and Sweden). Instead of offering instructions in the form of written posts, the livestreams were used to share information about online protests, announce the official hashtags, publicize local strike ‘themes’ (e.g., food waste, fast fashion, etc.) and ask participants to use the tag functions to increase visibility (e.g., geolocation, friends, politicians, other activist groups, etc.)

Not all FFFs approached the digital strike as a purely online tactic. In some countries, organizers launched campaigns blending digital and analogue strategies to make online actions visible to the public. Some examples of this are the hologram strike in Poland, where organizers projected former protest images onto government buildings; the shoe strike in Sweden, where protesters set up shoes to symbolize their physical presence or the balcony strikes in Austria, Hungary and Bulgaria, where followers displayed their protest signs outdoors. In some cases, these efforts created resonance with local media. In Germany, organizers coordinated a sign protest in front of the parliament, which was visually impactful and thus covered by various prestige news outlets.

#### Challenges

In addition to the digital strike, contentious actions such as online campaigns and ‘challenges’ were quickly put in place by local FFFs. For instance, 12 national collectives joined campaigns on the occasion of Earth Day 2020, asking followers to pledge reducing their energy use, go plastic free for the remainder of the month, or donate to local food banks. Lithuania launched a photo challenge, asking followers to take a picture from their window and describe what climate issues are visible from people’s homes. In Italy, where the pandemic struck forcefully early on, FFF activists organized a flashmob, where recorded themselves performing a dance choreography to the Bee Gees’ hit ‘Staying Alive’.

#### Online petitions

Organizers also employed two other classic contentious tactics – petitions and open letters, albeit scarcely. Topics included aviation bailouts (Austria, Lithuania) or asking local governments to stick to climate action plans. Belgium, Bulgaria, Denmark, France and Slovakia launched petitions with a focus on the EU. Six collectives also started petitions on local issues, including water supply (Italy); climate policy (France); railway construction (Latvia); pandemic mismanagement (Romania); environmental NGO regulation law (Slovenia) and the fossil fuel industry (Spain). Several online petitions were shared to the national Facebook pages from larger NGOs with a climate agenda, such as Greenpeace (Belgium) or the World Wildlife Fund (Hungary). FFF national collectives, however, would post external campaigns once without updating the results.

### Information and education

#### Webinars

Under the social distancing measures and public assembly restrictions, FFF organizers pursue components of their campaign beyond traditional striking. The production and circulation of educational/informative interactive content related to the cause constitutes a recurrent activist practice across almost half of the countries in our sample. During the early stage of the online phase, organizers have veered toward digital education formats, the most popular among them being the ‘webinar’. An online seminar, the webinar usually brings together experts on a topic related to FFF’s profile, while supporters of the movement are invited to join in order to expand their knowledge and understanding of the topic.

Webinars constitute a popular action repertoire employed by 15 out of 27 country groups. During the 10-week sampling period, Bulgaria leads with eight thematic webinars, followed by Germany with six. Themes ranged from topics that covered the pandemic, infectious diseases, global warming, and the pitfalls of capitalism, to name a few. Denmark also offered a training webinar for followers, teaching them how to write climate-focused news stories. Mostly, webinars were pre-recorded and streamed at a scheduled time. Importantly, FFF collectives across the EU organize their own educational events, while six national pages also featured the same, centralized ‘Talks for Future’ webinar put on by the FFF International hub, which featured Greta Thunberg on the topic of European political parties and their programs on climate change.

#### Live events

Next to (pre-recorded) webinars with experts, 14 national Facebook pages also featured educational livestreams with various programs, including a debate event (Denmark), an online conference with scientists (Italy) and interactive panels with climate experts (Czechia, Slovakia). Two livestreams engaged thematically with the pandemic itself (France, Italy), discussing the nexus between health and climate. A Q&A session with Greta Thunberg was also shared among several national collectives as a livestream (e.g., Germany).

#### Online news

19 national Facebook groups provided followers with online articles about climate change issues, such as fossil fuels and wildlife endangerment. Under the pandemic, many of them highlighted articles that made connections between the Corona crisis and the climate crisis, harnessing the mobilizing potential of media reporting. Shared articles discussed the EU-pandemic management (Estonia), the lack of crisis awareness (Finland), as well as the link between air pollution and health issues (Hungary, Italy, Latvia). Many used the COVID-19 crisis as a contentious frame to postulate that climate change accelerates pandemics (Lithuania, Slovakia), forecasting climate change as the next global pandemic (Germany, Portugal). The articles predominantly came from external news sources, though four national collectives (Austria, Denmark, Italy and Portugal) invested in in-house news production, offering their own reporting on climate issues.

### Community engagement

#### Thanking participants

FFF organizers employ digital tools to make participation in online protest and events visible. Denmark and Greece showcased livestream attendees and Zoom participants by sharing screenshots of attendees on their national pages. Importantly, this tactic was employed to reward strikers for their participation in the online strike replacement events. In mimicking the ‘bodies in the streets’ tactic of analogue protests, 11 countries featured a collage of digital strikers’ profile pictures with their homemade signs. Furthermore, FFF organizers put much effort into thanking followers for participating in the new digital strikes in the form of ‘thank you’ posts, which often included announcements for upcoming events and emphasized the hashtags #climatestrikeonline and #digitalstrike.

#### Leisure activities

Collectives also sought to keep spirits high and offer leisure activities. To boost morale, organizers shared messages relating to overcoming social isolation and maintaining solidary with the climate cause in the wake of the COVID-19 pandemic (Belgium, Denmark, Hungary, Italy and Lithuania). Correspondingly, Swedish organizers facilitated a weekly contest where followers could vote on their favorite protest sign. Leisure activities related to the movement and the cause, for instance in the form of a digital book club (Croatia), a climate-movie repository (Italy), co-playing videogames (Sweden) and movie nights (Czechia). Denmark and Spain offered a reprieve from the pandemic by asking followers to send in goofy letters or make climate-inspired art, while Belgium, Czechia, Italy and Lithuania streamed live concerts of activist-musicians on their Facebook pages.

### Partnership development^2^

#### Internal cross-promotion

The social distancing measures under COVID-19 freed up time and resources for organizers to engage groups within and outside of FFF. Inside of the movement, some national collectives showcased other FFF’s efforts. Germany, for instance, advertised a video by the local FFF collective in Berlin. Austria was the only country that regularly featured posts by satellite groups within the larger FFF youth movement, underscoring the allyship from Scientist for Future, Parents for Future, Artists for Future, and Doctors for Future. Bulgaria, Cyprus and Malta also shared some FFF International hub posts, although – overall – the cross-promotion within the movement was quite marginal.

#### External collaborations

17 out of 27 collectives used this time to expand ties with other activists and issue-proximate groups. As such, we see collaboration unfold as a core aspect of community building, evidenced in our data through the plethora of posts that sought to connect followers and sympathizers to the cause. Romania shared posts for a local ‘Polluters Out’ collective, Croatia boosted visibility for the national ‘Zero Waste Network’, Cyprus and Latvia featured a challenge by an NGO called ‘Fashion Revolution’, while Estonia and Latvia promoted a campaign by the ‘Rail Baltica’ initiative. Italy was the only national FFF collective to showcase an activity by the more ‘radical’ environmental group ‘Extinction Rebellion’. On a transnational level, Greenpeace campaigns were advertised to FFF Facebook followers in Bulgaria, Croatia and Hungary.

Notably, FFF organizers created posts connecting the movement to other causes that have climate and environmental dimensions, including indigenous rights or migration. On International Women’s Day, collectives made multiple posts addressing gender inequality as a roadblock for climate justice (Belgium, Italy, Malta, Portugal and Slovenia). With respect to the COVID-19 pandemic, Bulgaria and Croatia raised awareness on the impending unemployment crisis.

#### Media partnerships

The assembly restrictions and social distancing measures also created space for FFF national collectives to strike partnerships with media organizations, both to produce and to publicize content relating to organizational activities, the movement and the cause. Three cases stand out here, first and foremost Portugal, where national organizers developed a podcast series with coalitional partners, highlighting the intersectional dimensions of climate justice as they relate to queerness, migration, and race, to name a few. In Italy, a new partnership with the media organization LifeGate led to the production and cross-promotion of videos and online articles. Austrian organizers forged a partnership with the independent online magazine Klimareporter.de, collaborating on climate-related news and cross-sharing their original content.

In the next section, we discuss FFF’s digital action repertoire under Corona with respect to the move from analogue to digital, the blending of online and offline elements, and the spectrum of action between contention and collaboration.

## Discussion

The four Facebook action tactics that FFF organizers around the EU resorted to during the COVID-19 pandemic (see Figure 1) represent an example of ‘improvis[ing] on shared scripts’ (Tilly, 2006: 35). In the digital phase, FFF expands their movement’s flagship action, the “school strikes,” to social media. The resulting digital strikes and weekly challenges open up new avenues for creative participation (McFarland, 2010) by asking movement followers to engage in content production and by giving them the opportunity to personalize the movement’s message. The digital strike and weekly challenges both re-used and further legitimized a fairly ‘simple’ recipe for organization and participation: organizers provided participants with instructions on how to tailor a general message and publicize it on their own profile(s). In turn, participants made a sign, took a picture and set it as their profile picture or shared it on their social media channels. While this recipe builds upon the mobilizing and sharing appeal of personalizable connective action frames (Bennet and Segerberg, 2012), it can also foster a vision of climate change activism as an individual and low-cost gesture. The question here is how such a vision may undermine the movement’s long-term efforts to build collective identities and action frames.

Community-building, on the other hand, could also emerge as a side-effect of the digital strikes and weekly challenges. Just as FFF projects a community when followers gather on the street, the virtual co-presence on digital strike days or while attending webinars or livestreams, can afford a sense of community, even if on a small scale (Earl and Kimport, 2011). The subsequent posting of screenshot collages from live events as a token of gratitude for participation employs mediated co-presence to solidify the ‘imagined community’ in the digital space. Indeed, picture-sharing from strikes and particularly, of strikers, are commonly employed as mobilizing tactics in digital social movements (Theocharis et al., 2015).

Yet, forced digitalization as tactical innovation during crisis remains dialectical: it does not only provide new opportunities, but it also brings along new challenges that can affect the movement’s identity and action frames. In the case of community-building, for instance, this work seemed to be exclusively directed at existing supporters. In contrast to organizational social media uses (Lovejoy and Saxton, 2012), FFF national collectives did not appear to actively recruit new supporters or fundraise for the movement’s activities. In a few instances, organizers asked followers to donate to local projects to help with Corona-related impacts on the community. Only a few online challenges attempted recruitment in asking followers to tag a friend or share their message with someone in their immediate circle, despite the fact that member mobilization challenges could have easily been implemented. The esthetic of the digital strikes showcases the same recurring image of a young (often feminine performing) person holding a sign and smiling to the camera. For a movement like FFF, this raises questions on how digitalization can be best harnessed to further expand the movement on the long-term.

Another notable challenge brought along by forced digitalization was the loss of momentum in terms of contention. Contentious action is a key element of social movements (Rucht, 2017); it is notoriously costly – participation in school strikes, for instance, requires time, energy, mobility and funds – but performed contention commands attention from political elites and the public (Tarrow, 2011). In the case of FFF, the digitalization of their activist repertoire sidelines contentious engagement. While the digital strike format asked supporters to perform themselves as protesters, these events did not get noticed by political structures. Furthermore, partaking in a digital format made the symbolic yet powerful withdrawal of resources of a ‘strike’ unnecessary: FFF’s withdrawal of resources in the form of students skipping class as a disobedient act to get attention for their demands became purely performative. Digital strikers could share their strike images without this action directly impacting their school attendance.

We also found that during the digital phase in early 2020, contention yielded to internal community-building work. While FFF national collectives also employed challenges and online campaigns to prompt action by followers, these calls were often generic (e.g., 10 things you can do for the environment during the shutdown’) and difficult to follow-up on. Vague calls to action relied on the self-commitment of digital users pledging themselves to a particular activist gesture. This rendered the actual contentious dimension ambiguous (Kim and Kim, 2008).

Contentious action does not only disrupt everyday life and carry a symbolism that forces political elites to respond. It also provides opportunities for amplification via news media coverage. In the case of FFF, blended components that combined online and offline forms of action seemed to us more promising in being able to command attention outside of the movement’s social media network of followers. Six country organizers purposefully recovered physical elements of protests, employing organizational tactics that went beyond transplanting the analogue action to the virtual space, as evidenced in projecting holograms onto political landmarks or displaying signs in public spaces. Such blending still offers low-cost efforts for participants, though increasing work for organizers, who are now taking on the responsibility of moving online to offline (Van Laer and Van Aelst, 2010). While also showcasing creativity, blended events generated media attention, which is needed for activist publicity (Downey and Fenton, 2003). This is even more important for those FFF chapters with a rather modest online following (see Table 1). However, such blending remained rather rare across our sample.

One surprising empirical finding was the rather minimal transnational coordination visible across the Facebook pages of the FFF collectives across our sample. While some collectives amplified content from the FFF international hub (e.g., the Q&A with Greta Thunberg), this was not done consistently across the movement. The same minimal coordination concerned the new digital hashtags: while local hashtags are certainly useful, the use of some common hashtags can increase visibility on social media platforms (Sorce and Dumitrica, 2021). In fact, national collectives would, at times, forget to instruct digital participants to use international hashtags such #climatestrikeonline and #digitalstrike, thereby forfeiting local participants’ chances to have their posts rendered more visible. In addition, organizers rarely tagged stakeholders, such as politicians or news media, obscuring actions that remained online.

Finally, even though the majority of FFF collectives within the EU adopted the digital strike format, they did not cross-promote their events. Proximity often spurs interest and as decentralized movements such as FFF struggle with coordination between locations (Tormos-Aponte and Garcia-López, 2018), digitalization makes the fostering collaborations within the movement more convenient. This, we think, is even more important in the context of a movement that rallies around a crisis that knows no borders, such as climate change.

## Conclusion: What has the pandemic changed and what is here to stay?

To assess how forced digitalization affected FFF’s action repertoire under the restrictions brought about by the COVID-19 pandemic, we have employed a case study with qualitative content analysis of the movement’s online communication patterns on Facebook. Analytically, the movement’s forced digitalization resulted in four clusters of action tactics: digital contentious actions, online information and education, digital partnership development and online community engagement. The movement recombined existing formats from the physical action repertoire, such as the strike and educational events, rather than creating entirely novel tactics of action. Yet, the adaptation of the movement’s flagship tactic – the school strike – is accompanied by a loss of symbolic power, as ‘digital strikes’ no longer entail the withdrawal of resources as a form political resistance. More research is needed to understand how a simple digital strike recipe – make a sign, take a picture, post to social media – compares to the political impact of global masses in the streets. Future research could study the long-term effects of the pandemic-forced digitalization on the expansion and political impact of social movements such as FFF.

At the same time, the restriction to the online space afforded the movement opportunities to network and build community with existing followers and issue-proximate groups, making collaboration an interesting focus to study in the digitalization of social movements. Although contention took a back seat to collaboration, the interactive digital formats, such as livestreams and webinars showcase FFF’s timely ‘tactical flexibility’ (McCammon, 2003) that kept the movement alive under the COVID-19 restrictions. Different levels of professionalization across the chapters appear to mediate the FFF’s adaptation strategy, as the comparative dimension of our project revealed significant differences in the use of social media functionality and communication practices across the collectives (e.g., using recurring hashtags, tagging stakeholders, event promotion, multimedia formats, frequency of posting, etc.).

Crises create times of forced innovation, during which tactical flexibility and professionalization play a key role in the outcome of digital actions. While small innovations that recombine existing tactics are quite common (Wang and Soule, 2016), the political impact of FFF’s digital repertoire and its consequences for long-term mobilization remain to be seen. The intensification of affective work and the reliance on simple recipes for digital ‘contention’ represent, in our view, rather ambiguous transformations in the life-cycle of the movement. In that sense, the movement seemed to have shifted its emphasis from external political work to internal preservation.

The particular case we examine requires an important methodological consideration – while a qualitative content analysis allows the systematization of communication material as categories, the function of the elicited or desired responses by social media users is not as clear-cut in the context of social movements if the codes are ‘actions’. As noted by Milan and Beraldo (2019), categorizing activism and collective action in digital contexts must take into account the ‘overlap of…categories in the empirical world’ (8). In their own attempt to typologize data activism, the authors explain that the codes contained in their categories are action-based and as such, the real-life implications vary by user. Simply put, while a Facebook post asking followers to take part in a digital strike only fits one category (digital contentious action), the effect of this digital participation might be that users feel both connected to the cause (mobilization) *and* feel part of a group (community building). Hence, we make suggestions about the potential functions and implications of these posts on social media audiences, while measuring the actual effects remains open for examination.

Our study remains limited by its focus on a single platform as opposed to the larger communication ecosystem (Treré, 2019) in which FFF exists. Furthermore, we focus on FFF in the EU at the expense of other geographical locations which may have undergone different adaptation processes. In some cases, local FFF collectives may actually be more active than national hubs. Future research could build upon our typology through a cross-platform approach or examine how other activist groups have managed the balance between contention and collaboration during the pandemic, or study how different Corona-related policies impact the digitalization of activism in various countries.

We particularly want to signal the need to further examine whether such transformations are here to stay and whether other social movements underwent similar adaptations during the pandemic. In terms of wider implications for the transformation of activist repertoires during COVID-19, our study focused on the case of FFF, whose internal values promote compliance with science, which featured prominently into the movement’s swift rhetorical adaptation to the online space in compliance with virus containment efforts (Sorce and Dumitrica, 2021). Around our sampling period, other social movements, however, continued to protest in person (such as the worldwide Black Lives Matter protests). More empirical research on the different strategic choices and digital media uses is needed to study ensuing tactical transformations of social movements in times of crises.

## Notes

1. English, French, German, Italian, Portuguese, Romanian, Spanish, and Dutch. For languages beyond our skills, we relied on Facebook’s automatic translation function and double-checked unclear phrases with native speakers in our professional network.

2. We have opted to refer to this type of action as partnership development, rather than coalition building, as we also capture collaborations with media organizations. In social movement literature, coalition building mainly refers to making ties with other activists (Van Dyke and Amos, 2017); however, we see collaborations with media organizations as an important development in FFF’s digital partnership work.
